# Relationship between Choroidal Thickness and Visual Field Impairment in Acute Zonal Occult Outer Retinopathy

**DOI:** 10.1155/2017/2371032

**Published:** 2017-07-05

**Authors:** Yuki Hashimoto, Wataru Saito, Michiyuki Saito, Yuka Hasegawa, Akari Takita, Shohei Mori, Kousuke Noda, Susumu Ishida

**Affiliations:** ^1^Department of Ophthalmology, Faculty of Medicine and Graduate School of Medicine, Hokkaido University, Sapporo, Japan; ^2^Kaimeido Eye and Dental Clinic, Sapporo, Japan

## Abstract

**Purpose:**

To evaluate sequential changes in choroidal thickness at the affected area in patients with acute zonal occult outer retinopathy (AZOOR).

**Methods:**

This retrospective observational case series included 14 affected eyes and 6 unaffected fellow eyes from 10 AZOOR patients with impaired macular area. Using enhanced depth imaging optical coherence tomography, choroidal thicknesses at the subfovea and at nasal and temporal sites 1000 *μ*m away from the fovea were manually measured at baseline and 3 and 6 months thereafter. Changes in the choroidal thicknesses and the average threshold at the affected area on Humphrey perimetry were compared during the 6-month follow-up.

**Results:**

In AZOOR eyes, the average threshold at the affected area significantly increased over time, while outer retinal structure ameliorated. The mean choroidal thicknesses at all the sites measured significantly decreased at 3 and 6 months compared with baseline values in AZOOR eyes, but not in fellow eyes. There was an inverse correlation between the changing rates of the average threshold and the subfoveal choroidal thickness at 6 months from baseline.

**Conclusion:**

The current data suggest that choroidal thickness at AZOOR-affected area significantly decreased with regression of AZOOR and this anatomical change correlated with the functional recovery.

## 1. Introduction

Acute zonal occult outer retinopathy (AZOOR), first described by Gass in 1992, is an idiopathic syndrome with acute outer retinal impairment [1, 2]. Visual field defects in AZOOR patients are caused by outer retinal impairment and are demonstrated by decreased responses on multifocal electroretinography (ERG) [3] and disrupted ellipsoid zones detected by spectral domain optical coherence tomography (SD-OCT) [4, 5]. Fundus autofluorescence can also visualize retinal pigment epithelium (RPE) abnormalities in the affected area during the acute and chronic stages of AZOOR [6, 7].

The pathogenesis causing the photoreceptor impairment in AZOOR is still unresolved. Regarding the association between AZOOR and the choroid, abnormalities on indocyanine green angiography (ICGA) have been reported as hypofluorescence in areas related or unrelated to the lesions [8–13]. Moreover, we recently demonstrated using laser speckle flowgraphy (LSFG) that choroidal blood flow velocity at AZOOR lesions significantly increased as visual function and outer retinal structure improved [8]. Since the choriocapillaris supplies nutrients and oxygen to the photoreceptors, these observations suggest a correlation between choroidal circulatory impairment and the pathogenesis of AZOOR, although further studies are needed to verify the relationship [14].

There are only a few reports that evaluated choroidal thickness in AZOOR. Subfoveal choroidal thickness in AZOOR patients tended to be less than that in normal eyes without retinal diseases (243 *μ*m versus 289 *μ*m) [6], although there was no significant difference between the two groups. The tendency may have resulted from the higher frequency of myopia among AZOOR patients. In 4 patients with AZOOR associated with punctuate inner choroidopathy (PIC), central choroidal thickness decreased together with the recovery of outer retinal structure and visual function [15]. In the acute stage of PIC, however, increased choroid thickness was noted at the affected area, even when it was not complicated by AZOOR [16]. By contrast, it remains unknown how choroidal thickness alters during the clinical course of AZOOR.

The aim of this study was to evaluate sequential changes in choroidal thickness at the affected area in eyes with AZOOR.

## 2. Methods

### 2.1. Patients

This retrospective observational case series included 14 affected eyes and 6 unaffected fellow eyes as controls from 10 patients with AZOOR (2 men and 8 women) who visited Hokkaido University Hospital from May 2011 through September 2014. Inclusion criteria were AZOOR eyes showing outer retinal morphological abnormalities in the macular area on OCT at the initial visit, regardless of the types of scotomata, and receiving choroidal thickness measurements with enhanced depth imaging- (EDI-) OCT for up to 6 months after baseline. The current study was approved by the ethics committee of Hokkaido University Hospital and followed the tenets of the Declaration of Helsinki. Informed consent was obtained from each subject after the nature and potential consequences of the study had been explained.

### 2.2. Diagnosis

The diagnostic criteria for AZOOR were as follows: acute visual field or vision loss usually with concurrent photopsia; one or more visual field defect regions that could not be explained by funduscopic examination or fluorescein angiography (FA); decreased multifocal ERG responses corresponding to retinal sites with visual field defects; and outer retinal morphologic abnormalities, including absence or discontinuity of the ellipsoid zone and/or the interdigitation zone on OCT [2, 4, 5, 17]. Patients who showed funduscopic and angiographic findings consistent with white dots observed in AZOOR complex such as multiple evanescent white dot syndrome (MEWDS) were excluded.

### 2.3. Treatment

Of the 10 patients included in this study, 6 patients (9 eyes) who had the best-corrected visual acuity (BCVA) of more than 0.5 at the initial visit with nonprogressive clinical courses or who refused the administration of systemic corticosteroids despite an initially worsened BCVA were followed up without treatment (cases 1–6). Four patients (5 eyes) with an initially worsened BCVA and progressive central or paracentral visual function loss were treated with systemic corticosteroids, including corticosteroid pulse therapy or oral prednisolone (cases 7–10). The regimen for corticosteroid pulse therapy has been described previously [8]. In cases with worsening visual field defects on perimetry or worsening subjective symptoms during follow-up visits, the dose was temporarily increased.

### 2.4. Ophthalmic Examinations

At the initial visit, each patient underwent thorough ophthalmic examinations including BCVA, indirect ophthalmoscopy, FA, ICGA, 20 J single-flash ERG, and SD-OCT (cross-sectional retinal B-scans of 5 × 5 lines) combined with EDI-OCT (RS-3000 Advance; NIDEK, Gamagori, Japan). Several days later, these examinations were followed by visual field testing (Goldmann perimetry and a Humphrey 30-2 Swedish Interactive Threshold Algorithm (SITA) standard test), fundus autofluorescence, and multifocal ERG. BCVA, Humphrey perimetry, and OCT findings were assessed at baseline as well as at 3 and 6 months after baseline.

### 2.5. EDI-OCT

**Figure 1:**
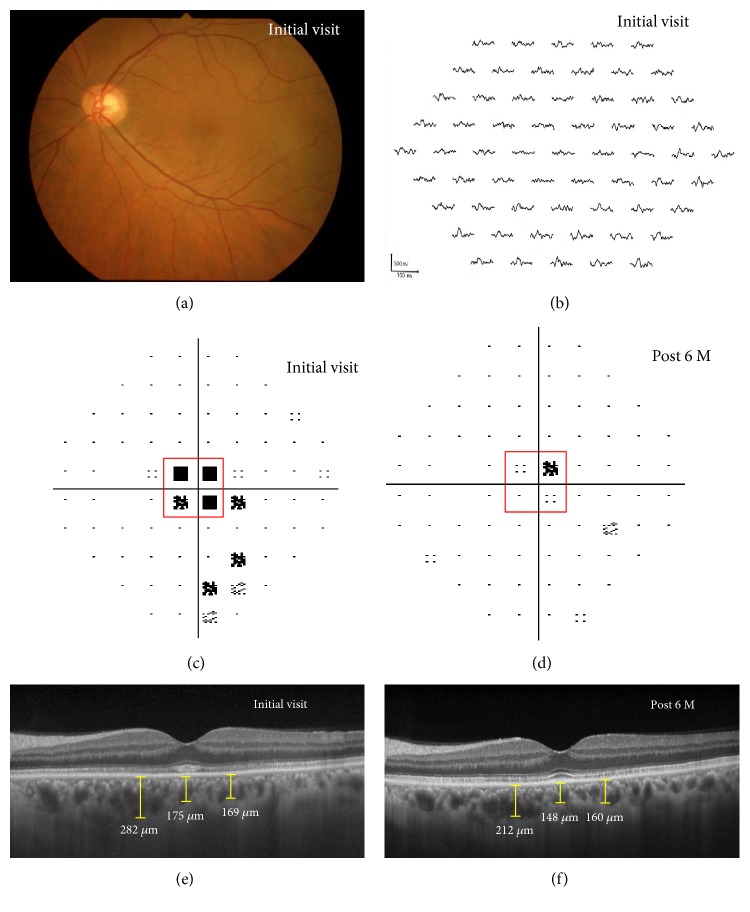
Photographs of the left eye at the initial visit (a, b, c, and e) and 6 months after the start of systemic corticosteroids therapy (d, f) in a patient (case 9) with acute zonal occult outer retinopathy (AZOOR). (a) Fundus photograph showing a normal retinal appearance. Best-corrected visual acuity (BCVA) was 1.0. (b) Multifocal electroretinography showing a decreased amplitude at the retinal site corresponding to the visual field defect (Figure 1(c)). (c) Humphrey perimetry showing decreased sensitivity within a central 30° area. (d) Visual field impairment markedly improved. The BCVA improved to 1.5. (e) Enhanced depth imaging optical coherence tomography (EDI-OCT) revealing the loss of the ellipsoid zone at the macula. Choroidal thicknesses at the subfovea and nasal and temporal sites 1000 *μ*m away from the subfovea were 175, 282, and 169 *μ*m, respectively. (f) The choroidal thicknesses at all sites decreased to 148, 212, and 160 *μ*m, respectively, with the recovery of the macular ellipsoid zone.

EDI-OCT measurements were obtained for each of the evaluation points at the initial visit and 3 and 6 months after the initial visit for nontreated AZOOR patients and before treatment and 3 and 6 months after the start of treatment for AZOOR patients receiving systemic corticosteroids. Using a horizontal scan through the fovea (scan length, 9.0 mm), choroidal thicknesses at the subfovea and at nasal and temporal sites 1000 *μ*m away from the subfovea were determined by manually measuring the distance from the outer border of the hyperreflective line corresponding to the RPE to the outer border of the choroid in 14 AZOOR eyes and 6 unaffected fellow eyes (Figures 1(e) and 1(f)). Three authors (Yuki H., Yuka H., and A.T.), who were blinded to the subjects' clinical information, independently examined EDI-OCT images. We assessed the statistical significance of differences in average choroidal thickness values between stages.

### 2.6. Visual Function

Using Humphrey perimetry, 4 adjacent threshold points at the lesion area were selected at the lesion area in the AZOOR eyes (Figures 1(c) and 1(d); red squares) and the corresponding area to the 4 points evaluated for each affected eye in the fellow eyes. The average threshold was calculated as the numerical index of changes in visual field impairment.

### 2.7. Statistics

All results are expressed as the mean ± standard deviation (SD). BCVA was converted to the logarithm of the minimum angle of resolution (logMAR) scale for the purpose of statistical analyses. Mann–Whitney *U* test was used to examine the differences in refractive error and choroidal thickness between the AZOOR and unaffected fellow eyes. The Friedman test and Scheffe's paired comparison test were used to examine sequential changes in BCVA, the average threshold on Humphrey perimetry, and the choroidal thicknesses. Spearman's rank correlation coefficient was used to examine a correlation between subfoveal choroidal thickness (SCT) and average threshold. For all tests, *P* values less than 0.05 were considered statistically significant.

## 3. Results

### 3.1. Patient Demographics

Table 1 shows the clinical characteristics of AZOOR patients included in this study. The mean age was 40.6 ± 14.1 years ranging from 17 to 64 years. The mean follow-up duration was 22.7 ± 11.3 months ranging from 6 to 39 months. Six patients had unilateral involvement at the initial visit, and no unaffected fellow eye developed signs of AZOOR during follow-up. The presumed duration from AZOOR onset to the first visit to our hospital was the mean 1.8 ± 1.8 months. The mean refractive error was −4.4 ± 3.9 D ranging from −14.25 D to +0.75 D in the AZOOR eyes (*n* = 14) and −3.2 ± 5.0 D ranging from −14.25 D to +0.25 D in the unaffected fellow eyes (*n* = 6). AZOOR eyes tended to be more myopic than fellow eyes, but there was no significant difference in refractive error between the two groups (*P* = 0.34).

### 3.2. Ophthalmic Findings

At the initial visit, the retina was funduscopically normal in 12 of 14 eyes with AZOOR (Figure 1(a), Table 1). The recurrence of AZOOR was not observed in any of the 14 affected eyes within the 6-month follow-up period but in 3 eyes thereafter up to their final visits. None of the 12 eyes with normal retinal appearance at the initial visit demonstrated retinal atrophy thereafter. In 11 eyes examined, single-flash ERG showed a normal amplitude in 7 eyes, reduced a-wave amplitude in 3 eyes, and reduced b-wave amplitude in 1 eye. There were noticeably reduced multifocal ERG responses corresponding to visual field loss in all affected eyes (Figure 1(b)). Of the 8 eyes examined, fundus autofluorescence showed temporary hyper-autofluorescence in 5 eyes and normal appearance in 3 eyes at the initial visit, corresponding to sites with the visual field impairments.

### 3.3. Fundus Angiography

FA showed a normal appearance in 10 eyes (62.5%), retinal vascular wall staining with leakage in 2 eyes (12.5%), and optic disc staining in 2 eyes (12.5%). On ICGA, 4 eyes (28.5%) appeared normal, but patchy hypofluorescence in the macular area or midperiphery was observed in 7 eyes (50.0%). In addition, 7 eyes (50.0%) had diffuse choroidal hyperfluorescence from the posterior pole to the midperipheral region during the middle phase.

### 3.4. Retinal Morphology

The ellipsoid zone in the AZOOR-affected area was invisible in 3 eyes (21.4%), discontinuous in 8 eyes (57.2%), and normal in 3 eyes (21.4%) at the initial visit (Table 1, Figure 1(e)), whereas the interdigitation zone was discontinuous or invisible in all 14 eyes (100%) at the initial visit. Six months after baseline, the ellipsoid zone was invisible in 0 eyes (0.0%), discontinuous in 3 eyes (21.4%), and normal in 11 eyes (78.6%) (Figure 1(f)), while the interdigitation zone was normal in 2 eyes (14.2%), discontinuous in 7 eyes (50.0%), and invisible in 5 eyes (35.8%).

### 3.5. Changes in Visual Function

Changes in BCVA and the average thresholds at the affected area on Humphrey perimetry in eyes with AZOOR (*n* = 14) are shown in Table 2. In nontreated eyes (*n* = 9, cases 1–6), the mean logMAR BCVA showed no significant improvement during the 6-month follow-up period (Friedman's test, *P* = 0.25). In corticosteroid-treated eyes (*n* = 5, cases 7–10), the BCVA at 3 and 6 months tended to improve compared with the baseline value.

**Figure 2:**
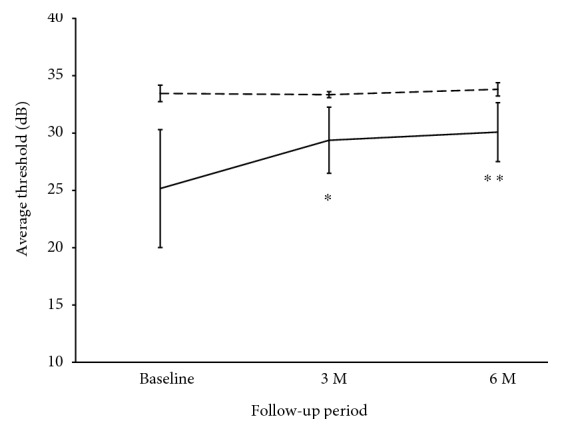
Changes in average threshold on Humphrey perimetry in eyes with AZOOR and unaffected fellow eyes. The “average threshold” is defined as the average values of 4 threshold points surrounded by a red square shown in Figures 1(c) and 1(d) at the AZOOR lesion area. In the AZOOR eyes (*solid black line*), the mean average threshold was significantly higher at 3 and 6 months than at baseline (^∗^*P* < 0.05, ^∗∗^*P* < 0.01, resp.), whereas it was unaltered in the fellow eyes (*dotted line*).

In the AZOOR eyes, the mean average thresholds in the affected area at 3 and 6 months were significantly higher than those at baseline (Figure 2, Friedman's test, *P* = 0.0008, and Scheffe's paired comparison test, *P* = 0.03 and *P* = 0.001, resp.). Similarly, in the nontreated eyes with AZOOR, the values at 6 months were significantly higher than at baseline (Table 2, Friedman's test, *P* = 0.03; Scheffe's paired comparison test, *P* = 0.03). Also in the corticosteroid-treated eyes, the average threshold showed the same trend. In contrast, the unaffected fellow eyes showed no significant change at 6 months in the average threshold (Figure 2, *P* = 0.60).

### 3.6. Changes in Choroidal Thickness

**Figure 3:**
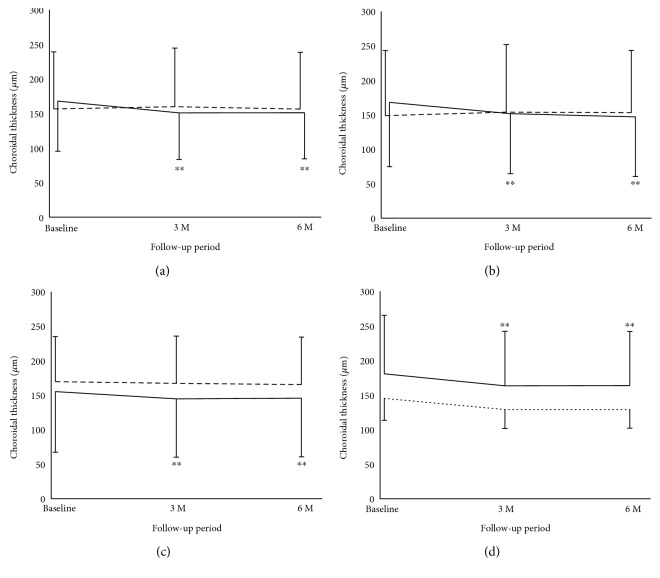
Changes in choroidal thicknesses using EDI-OCT in eyes with AZOOR and unaffected fellow eyes. (a) In the AZOOR eyes (*solid black line*), the mean subfoveal choroidal thickness (SCT) was significantly reduced at 3 and 6 months compared with baseline (^∗∗^*P* < 0.01, for both), whereas it was unaltered in the unaffected fellow eyes (*dotted line*). (b) In the AZOOR eyes (*solid black line*), the mean choroidal thickness at the nasal site 1000 *μ*m away from the subfovea was significantly reduced at 3 and 6 months compared with baseline (^∗∗^*P* < 0.01, for both), whereas it was unaltered in the unaffected fellow eyes (*dotted line*). (c) In the AZOOR eyes (*solid black line*), the mean choroidal thickness at temporal site 1000 *μ*m away from the subfovea was significantly reduced at 3 and 6 months compared with baseline (^∗∗^*P* < 0.01, for both), whereas it was unaltered in the unaffected fellow eyes (*dotted line*). (d) Similarly, in the nontreated AZOOR eyes (*solid black line*), the SCT significantly decreased over time compared with baseline (^∗∗^*P* < 0.01, for both). In the corticosteroid-treated eyes, it showed the same trend (*dotted line*).

Choroidal thickness changes in the AZOOR eyes and fellow eyes are shown in Tables 2 and 3. In all AZOOR eyes, the SCT values at 6 months were lower than baseline values. The SCT values at 3 and 6 months were significantly lower than baseline (Figure 3(a), Friedman's test, *P* < 0.0001; Scheffe's paired comparison test, *P* = 0.0004, *P* = 0.0002, resp.). In the unaffected fellow eyes, the mean SCT was unaltered during the 6 month follow-up period (Figure 3(a), *P* = 0.58). The SCT in the AZOOR eyes was 11.4 *μ*m more than in the fellow eyes at baseline, although there was no significant difference between values in two groups (*P* = 1.00; Figure 3(a)).

Similarly, the choroidal thicknesses at the nasal and temporal sites of the subfovea at 3 and 6 months were significantly lower than baseline values (Table 3, Figures 3(b) and 3(c), Friedman's test, *P* = 0.0001, *P* = 0.004; Scheffe's paired comparison test, *P* = 0.009 and *P* = 0.01, *P* = 0.0001 and *P* = 0.01, resp.). In the unaffected fellow eyes, the thicknesses at the nasal and temporal sites of the subfovea were unaltered during the 6-month follow-up period (Table 3, Figures 3(b) and 3(c), *P* = 0.58, *P* = 0.21, resp.).

In the nontreated eyes with AZOOR, values at 3 and 6 months were significantly decreased compared with those at baseline (Figure 3(d), Friedman's test, *P* = 0.001; Scheffe's paired comparison test, *P* = 0.006, *P* = 0.003, resp.). In the corticosteroid-treated eyes as well, the changes in SCT showed the same trend (Figure 3(d)).

### 3.7. Correlation between SCT and Visual Function

**Figure 4:**
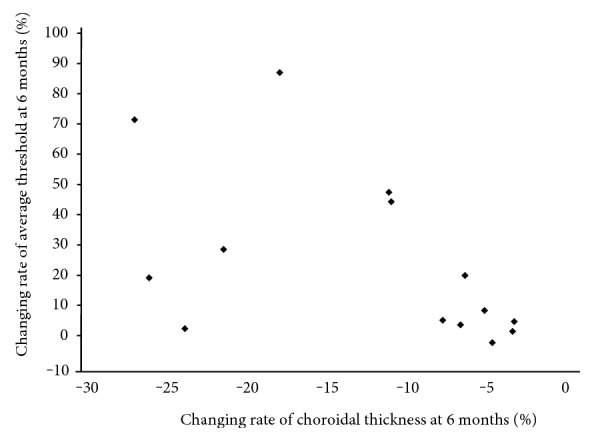
There was a significant inverse correlation between the changing rates of the average threshold and the SCT during the 6-month follow-up period in AZOOR eyes (*R* = −0.57, *P* = 0.03).

In the AZOOR eyes, there was a significant inverse correlation between the changing rates of the average threshold and the SCT at 6 months from baseline (Figure 4, *R* = −0.57, *P* = 0.03). No significant difference was found between changes in the SCT and logMAR values of the BCVA during the 6-month follow-up period (*R* = 0.36, *P* = 0.19).

## 4. Discussion

In the present study, by using EDI-OCT during follow-up for eyes with AZOOR, we made the following observations: (1) In both nontreated and corticosteroid-treated AZOOR eyes, the mean average threshold on perimetry in the affected area was significantly increased at 6 months after baseline, with improvement in outer retinal structures, whereas it did not in the unaffected fellow eyes. (2) In the AZOOR eyes, the macular choroidal thicknesses significantly decreased over time compared with baseline, whereas it did not in the unaffected fellow eyes. (3) There was a significant inverse correlation between the changing rates of the average threshold and SCT from baseline to 6 months in the AZOOR eyes.

In the present study, choroidal thickness in AZOOR lesion area significantly decreased with regression of the disease, with a significant correlation between the changes in the visual field impairment and SCT. These results were similar with our previous observations in MEWDS [18]. As concerns the mechanism underlying the sequential reduction in choroidal thickness in AZOOR eyes, two possibilities are speculated as follows: choroidal thinning (permanent volume loss) during the clinical course or transient choroidal thickening at the acute stage. Convalescent tissue loss is theorized to result from atrophic changes due to disease activity, and this is actually the case with some severe cases of AZOOR presenting progressive chorioretinal degeneration over time. However, the present AZOOR eyes in our case series exhibited significant functional improvements in visual field without the development or progression of any funduscopically visible chorioretinal atrophy during the 6-month follow-up period, in accordance with our recent report showing that visual prognosis in Japanese patients with AZOOR was better than that in Caucasian patients [12]. Moreover, there were a correlation between the extents of the choroidal thickness reduction and improvement of the visual field defects. These results may not be consistent with the speculation that AZOOR-lesion area in our cases developed choroidal atrophy during follow-up.

In the present study, there were no significant differences in choroidal thicknesses between the AZOOR eyes and the fellow eyes at baseline, although the mean SCT was 11 *μ*m more in the AZOOR eyes than in the fellow eyes at baseline. However, the baseline thickness values of the AZOOR eyes might be relatively underestimated, because the degree of myopia tended to be greater in the AZOOR eyes (−4.4 D) than in the control eyes (−3.2 D). Therefore, further studies using healthy subjects with a spherical equivalent matched are needed to examine whether the choroid in AZOOR eyes thickens in the acute stage.

In choroiditis such as Vogt-Koyanagi-Harada disease and serpiginous choroiditis, choroidal thickness decreased and choroidal blood flow velocity increased with regression of these diseases [19, 20], consistent with the inflammatory swelling of the choroid with blood flow disruption at the acute stage. These changes in thickness and circulation are therefore called an “inflammatory” pattern in the choroid [19, 20] and also seen in chorioretinal diseases with yet unidentified pathogenesis, such as acute posterior multifocal placoid pigment epitheliopathy or unilateral acute idiopathic maculopathy [21, 22]. Interestingly, this is true with our present (thickness) and previous (circulation) results [8] on AZOOR. The AZOOR complex is a syndrome with outer retinal impairment and includes MEWDS, PIC, acute macular neuroretinopathy (AMN), and multifocal choroiditis and panuveitis other than AZOOR [1, 2]. It has been reported that choroidal morphologic and circulatory changes in MEWDS, PIC, and AMN showed a similar pattern to AZOOR [18, 23–25]. Taken together, inflammation-related transient choroidal thickening with impaired circulation may be regarded as the common pathogenesis of the AZOOR complex at the active stage.

The primary limitations of our study include its retrospective design and the small sample size. In this study, choroidal thickness was manually measured using EDI-OCT B-scan. To overcome this measurement bias, further studies are needed to automatically measure choroidal thickness and volume by using swept-source OCT C-scan. Because of rarity of this disease, both eyes per one patient were used from 4 bilaterally affected cases; however, the statistical significance in SCT changes proved to be still maintained even if only the left eyes from 8 cases (other than cases 7 and 8) were analyzed to avoid any systemic confounders (Friedman's test, *P* = 0.002; Scheffe's paired comparison test, *P* = 0.01, *P* = 0.006, resp.).

## 5. Conclusions

In AZOOR eyes, choroidal thickness in AZOOR-affected area significantly decreased together with improvements in visual function and outer retinal structure. Furthermore, there was a significant correlation between reduction of choroidal thickness and improvement of visual field defects. These results suggest that choroidal thickness increases at the acute stage of AZOOR. Future studies are needed to further examine the involvement of choroidal thickening in the pathogenesis of AZOOR.

## Figures and Tables

**Table 1:** Clinical characteristics of patients with acute zonal occult outer retinopathy (AZOOR).

Case	Age	Sex	Eye	Medical or ocular history	Duration from onset to first visit (M)	Follow-up duration (M)	Refraction (D)	Goldmann perimetry	Funduscopic findings	Ellipsoid zone		Interdigitation zone	
										Baseline	6 M	Baseline	6 M
1	45	F	L	Hashimoto's disease	4	35	−14.25	CS + BSE + RS	Myopic macular CRA	−	+	−	±
2	64	M	R	None	6	30	−5.75	ND	Normal	±	±	−	−
			L				−6.00	ND	Normal	−	±	−	±
3	45	F	L	None	0.25	28	0.00	BSE + SAS	Normal	+	+	±	±
4	28	F	R	Depression	3	21	−5.00	PCC	Normal	+	+	±	+
			L				−5.25	PCC + IS	Normal	+	+	±	+
5	47	F	L	Bronchial asthma	2	16	−0.75	CS + BSE + IS + SAS	Normal	−	+	−	−
6	17	F	R	Hashimoto's disease	0.25	6	−6.75	BSE	Normal	±	+	−	+
			L				−6.75	BSE + CS	Normal	±	+	−	+
7	21	F	R	None	0.25	39	−7.50	CS + BSE	Morning glory optic disc	±	+	−	±
8	39	F	R	Basedow disease	2	33	−1.25	CS + BSE + IS	Normal	±	±	−	±
9	57	M	R	None	0.25	12	0.50	CS + BSE + IS	Normal	±	+	−	±
			L				0.75	CS + BSE + IS	Normal	±	+	±	+
10	43	F	L	None	0.50	7	−4.25	BSE + STAS	Peripapillary CRA	±	+	−	±

BSE: blind spot enlargement; CRA: chorioretinal atrophy; CS: central scotoma: IS: isolated scotoma; ND: not done; PCC: peripheral concentric contraction; RS: ring scotoma; SAS: superior arcuate scotoma; SIAS: superior and inferior arcuate scotoma; SNAS: superotemporal arcuate scotoma.

**Table 2:** Changes in visual functions and subfoveal choroidal thickness in AZOOR and fellow eyes.

Case	Treatment	Best-corrected visual acuity (logMAR)			Average threshold (dB)			Subfoveal choroidal thickness (*μ*m)					
								AZOOR eyes			Fellow eyes		
		Baseline	3 M	6 M	Baseline	3 M	6 M	Baseline	3 M	6 M	Baseline	3 M	6 M
1	None	0.3	−0.1	−0.1	15.5	30.5	29.0	45	41	37	33	20	33
2	None	0	−0.1	0	31.2	33.2	32.7	144	123	140	—	—	—
		−0.1	0.05	0	31.7	32.2	31.0	177	156	169	—	—	—
3	None	−0.1	0	0	23.2	33.2	33.5	284	251	255	293	280	284
4	None	0.1	0	0	28.7	28.5	30.2	276	255	255	—	—	—
		0.1	0.1	0	20.0	28.0	29.5	310	289	276	—	—	—
5	None	1	0.15	−0.1	17.2	28.2	29.5	123	99	90	152	169	144
6	None	−0.1	−0.1	−0.2	32.7	32.5	33.2	152	148	144	—	—	—
		0.05	−0.1	−0.1	26.0	30.2	31.2	115	107	107	—	—	—
Mean ± SD		0.13 ± 0.32	−0.01 ± 0.09	0.05 ± 0.06	25.1 ± 6.1	30.7 ± 2.0	30.8 ± 1.6	180.6 ± 84.7	163.2 ± 78.9	163.6 ± 78.1	159.3 ± 106.2	156.3 ± 106.5	153.6 ± 102.6
7	Steroid pulse	0.3	0.05	0.05	21.7	22.7	22.5	123	107	115	198	198	189
8	Steroid pulse	0.7	0.4	0.7	27.2	28.5	29.5	160	152	152	177	206	206
9	Steroid pulse	−0.2	−0.1	−0.1	28.5	29.5	29.2	173	156	148	—	—	—
		0	0	−0.2	26.0	29.2	31.0	175	144	148	—	—	—
10	Oral PSL, STTA	−0.1	0	0.05	22.7	25.2	29.2	94	86	82	86	86	82
Mean ± SD		0.14 ± 0.32	0.07 ± 0.17	0.10 ± 0.31	25.2 ± 2.6	26.9 ± 2.5	28.2 ± 2.9	145.0 ± 31.6	129.0 ± 27.6	129.0 ± 27.0	153.6 ± 48.6	163.3 ± 54.7	159.0 ± 54.8
Total		0.13 ± 0.32	0.01 ± 0.13	−0.01 ± 0.20	25.1 ± 5.1	29.3 ± 2.8	30.0 ± 2.5	167.9 ± 72.6	151.0 ± 67.4	151.2 ± 66.8	156.5 ± 82.6	159.8 ± 84.7	156.3 ± 82.3

Steroid pulse: corticosteroid pulse therapy; PSL: prednisolone; STTA: posterior sub-Tenon injection of triamcinolone acetonide.

**Table 3:** Changes in choroidal thicknesses at the sites 1000 *μ*m away from the subfovea in AZOOR and fellow eyes.

	Choroidal thickness (*μ*m)											
	Nasal site of the subfovea						Temporal site of the subfovea					
	AZOOR eyes			Fellow eyes			AZOOR eyes			Fellow eyes		
	Baseline	3 M	6 M	Baseline	3 M	6 M	Baseline	3 M	6 M	Baseline	3 M	6 M
1	43	43	41	41	35	39	26	20	20	24	26	24
2	103	82	84				134	99	115			
	61	57	57				173	169	169			
3	328	295	299	328	328	315	295	278	284	286	270	264
4	288	274	272				202	197	202			
	301	301	295				247	253	247			
5	216	193	177	127	167	136	152	127	117	220	220	210
6	123	123	119				156	165	148			
	138	125	115				152	148	138			
Mean ± SD	177.8 ± 101.8	165.8 ± 97.0	162.1 ± 96.9	165.3 ± 120.2	176.6 ± 119.8	163.3 ± 114.3	170.7 ± 71.0	161.7 ± 73.4	160.0 ± 73.7	176.6 ± 111.2	172.0 ± 105.2	166.0 ± 102.8
7	76	66	74	193	205	206	167	152	189	220	220	235
8	142	138	140	140	134	150	146	140	123	177	181	175
9	156	127	111				84	68	63			
	282	224	212				169	156	160			
10	94	74	61	63	53	72	70	53	63	90	86	84
Mean ± SD	150.0 ± 72.2	125.8 ± 56.6	119.6 ± 53.9	132.0 ± 53.3	130.6 ± 62.0	142.6 ± 54.9	127.2 ± 42.0	113.8 ± 44.0	119.6 ± 50.7	162.3 ± 54.0	162.3 ± 56.2	164.6 ± 62.0
Total	167.9 ± 93.3	151.6 ± 87.0	146.9 ± 86.5	148.7 ± 94.4	153.7 ± 98.1	153.0 ± 90.2	155.2 ± 65.6	144.7 ± 68.5	145.6 ± 69.0	169.5 ± 87.7	167.1 ± 84.5	165.3 ± 84.9
